# Role of microenvironmental periostin in pancreatic cancer progression

**DOI:** 10.18632/oncotarget.11533

**Published:** 2016-08-23

**Authors:** Yang Liu, Fan Li, Feng Gao, Lingxi Xing, Peng Qin, Xingxin Liang, Jiajie Zhang, Xiaohui Qiao, Lizhou Lin, Qian Zhao, Lianfang Du

**Affiliations:** ^1^ Department of Ultrasound, Shanghai General Hospital, Shanghai Jiaotong University, School of Medicine, Shanghai 200080, China; ^2^ Department of Instrument Science and Engineering, Shanghai Jiao Tong University, Shanghai 200240, China; ^3^ Department of Pathophysiology, Key Laboratory of Cell Differentiation and Apoptosis and National Ministry of Education, Shanghai Jiaotong University School of Medicine, Shanghai 200025, China

**Keywords:** periostin, pancreatic cancer, microenvironment, pancreatic stellate cells, EGFR

## Abstract

Pancreatic ductal adenocarcinoma (PDAC) is characterized by a prominent desmoplastic reaction. Pancreatic stellate cells (PSCs) are the principal effector cells responsible for stroma production. Aberrant up-regulation of periostin expression has been reported in activated PSCs. In this study, we investigated the role of periostin and the mechanisms underlying its aberrant upregulation in PDAC. We used lentiviral shRNA and human recombinant periostin protein to down and up regulate periostin expression *in vitro*. Specific oncogenic signaling pathways such as EGFR-Akt and EGFR-Erk-c-Myc were assessed *in vitro* and *in vivo*. Tissue microarray immunohistochemical assays including 80 pancreatic cancer tissues and paired normal tissues were used to understand the function relationship between periostin expression and PDAC pathologic stage and overall survival. We found that periostin was strongly expressed in PSCs and the stroma of PDAC tumors. We also observed a significant decrease in proliferation, metastasis, and clonality of pancreatic cancer cells when co-cultured with supernatant of periostin shRNA-transfected PSCs. Specifically, the biological behavior of periostin correlated with EGFR-Akt and EGER-Erk-c-Myc signaling pathways. Moreover, increased periostin expression significantly associated with advanced disease stage and decreased survival rate in PDAC patients. Together, our findings provide novel insights into the role of microenvironmental periostin in pancreatic cancer progression, and periostin may serve as a prognostic biomarker for PDAC.

## INTRODUCTION

Pancreatic ductal adenocarcinoma (PDAC) is a highly aggressive malignancy, with an overall 5-year survival rate of less than 5% and median survival period of less than 6 months [1–3]. Recently, the tumor microenvironment in PDAC has received increased attention, and is now recognized to be more than merely a passive bystander or host barrier against tumor progression [4, 5]. Rather, the cancer microenvironment of PDAC is characterized by a prominent desmoplastic reaction with a stromal content that is greater than the epithelial component, and is known to not only initiate and promote tumorigenesis but also mediate chemotherapy resistance [6, 7]. Moreover, pancreatic cancer cells (PCCs) exploit the tumor-supportive microenvironment [8]. The stroma is a dynamic cellular milieu that is mainly composed of pancreatic stellate cells (PSCs), fibroblasts, inflammatory cells, stem cells, and extracellular matrix as well as multitudinous cytokines and growth factors that can directly or indirectly interact with PCCs and change their biological behavior, thus playing a critical role in this rapidly progressive disease [9–11].

PSCs account for approximately 4% of the normal pancreas resident cells and are considered to be critical for the development of the pancreatic cancer desmoplastic response [8, 12]. When the pancreas is injured or becomes cancerous, a variety of factors induce PSC activation. The typical feature of the PDAC desmoplastic reaction is transformation of quiescent PSCs into activated PSCs [8]. Once stimulated by tumor cells, PSCs will perpetually be activated and produce excessive extracellular matrix to infiltrate and envelop the normal parenchyma via an autocrine periostin loop, creating a tumor-supportive microenvironment even under conditions of serum deprivation and hypoxia [6].

Periostin is a 90-kilodalton secretory protein originally identified as an osteoblast-specific factor that is preferentially expressed in the periosteum and functions as a cell adhesion molecule [6, 13]. Recently, periostin expression has been implicated in various types of cancer, including PDAC [14–17]. Periostin expression is 42-fold higher in PDAC compared with the normal pancreas at the mRNA level [1]. Tumor metastasis is the final phase of tumor progression. Periostin has been reported to promote the metastatic growth of colon cancer [18, 19] and the question of whether periostin can induce PCCs to the state of metastatic growth has attracted great attention [20]. Periostin was also identified as a potentially promising candidate for PDAC pathogenesis and is associated with a variety of signaling pathways that regulate cell activity [21].

Epidermal growth factor receptor (EGFR) is overexpressed in pancreatic cancer [22, 23]. We speculated that phosphorylation of EGFR mediated by periostin may initiate a downstream signaling cascade involving pathways such as Akt and Erk-c-Myc, which are implicated in carcinogenesis through their effects on cell proliferation, survival, metastasis, and gene expression. Current clinical efforts are directed toward studies involving inhibitors of EGFR-Erk signaling such as Erlotinib and SCH772984 [24–26]. However, the pathogenic mechanisms that regulate the biological behavior of PDAC remain elusive and should be reassessed in an unbiased manner [27, 28]. Moreover, the biological and clinical roles of periostin in PDAC are poorly described and the recent literature reports conflicting data [9, 14, 20]. Therefore, clarification of the periostin function and its related signaling pathways may help the development of new therapeutic strategies for PDAC.

Our findings not only identify periostin as a previously unrecognized PDAC microenvironment factor, but also clarify the potential role of periostin in PSC–PCC interactions and related signaling pathways downstream of EGFR in PDAC development and progression. We also demonstrate a correlation between periostin expression and patient survival. Collectively, the results of these studies verify that periostin has a pivotal role in PDAC progression and implicate it as a potential biomarker in this cancer. Furthermore, early therapy targeting EGFR pathways might exert an inhibitory effect on PDAC.

## RESULTS

### Periostin is produced exclusively by activated PSCs and is upregulated in human pancreatic cancer

**Figure 1:**
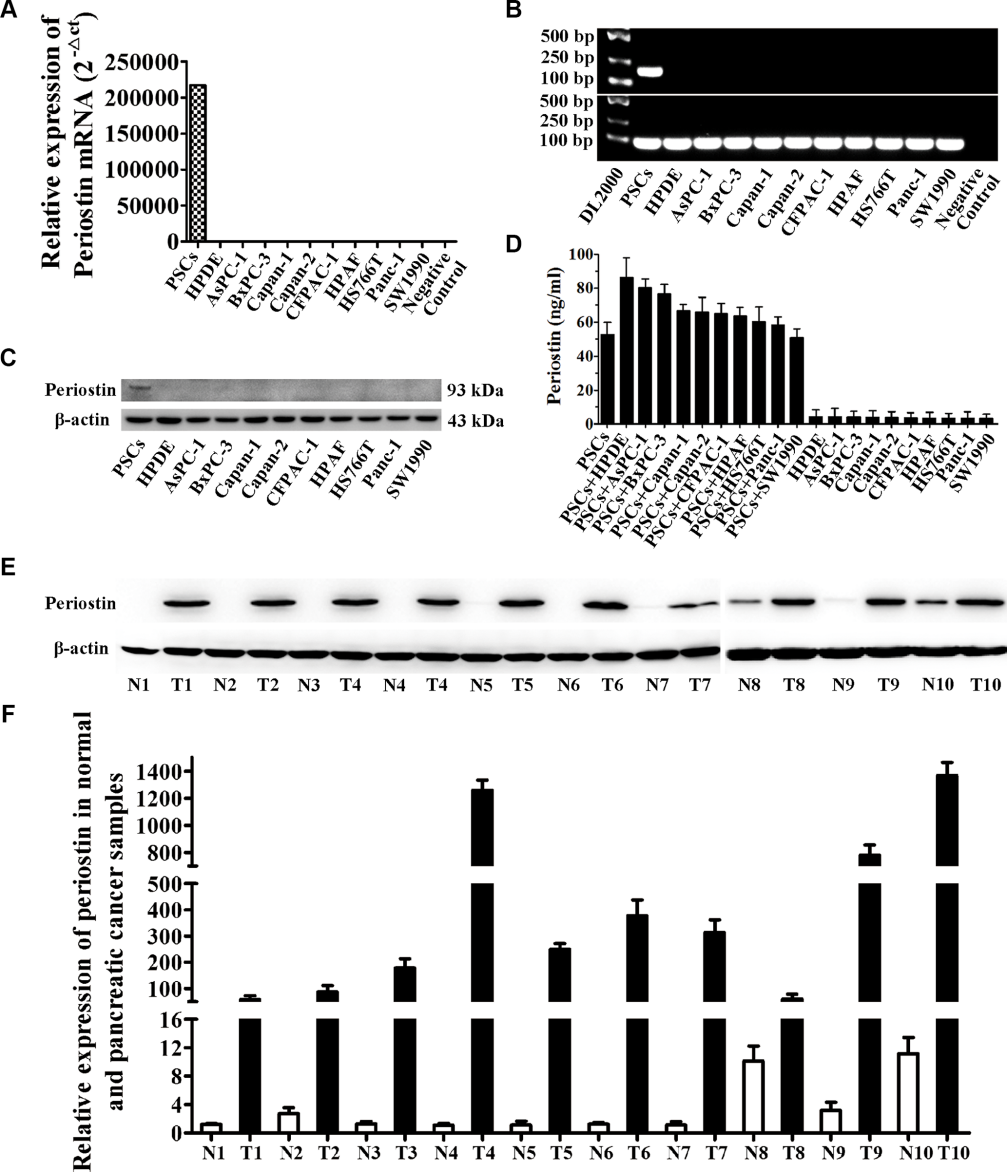
Periostin is exclusively expressed in PSCs and PDAC stroma (**A**) Quantitative analysis of periostin expression by real-time PCR in pancreatic stroma cell (PSCs), normal human pancreatic duct epithelial cells (HPDEs), and nine pancreatic cancer cell lines (PCCs). (**B**) Periostin transcript levels were examined by RT-PCR. The negative control indicates no template in the reaction. (**C**) Expression of periostin protein in these cell lines was determined by western blotting. (**D**) The level of secreted periostin was quantified by ELISA in PSCs and PCCs with or without co-culture. (**E**) Western blot analysis showed higher protein levels of periostin in tumor samples compared with the respective matched normal tissues (N, normal tissue; T, tumor). (**F**) Periostin mRNA expression level in 10 paired tumor samples and normal tissues.

To determine the significance of periostin in pancreatic cancer, we first analyzed the expression level of periostin in PCCs and PSCs by real-time RT-PCR, western blot analysis, and ELISA (Figure 1A, 1B, 1C and 1D). These results verified that periostin was exclusively expressed by activated PSCs. Moreover, PCCs could stimulate secretion of periostin by PSCs by co-culture (Figure 1D). We further performed western blot and real-time RT-PCR analyses on human pancreatic cancer samples and their respective paired normal tissues. All the tumor samples showed increased protein and mRNA levels of periostin compared with matched normal tissues (Figure 1E and 1F).

### Elevated periostin expression is associated with advanced pathologic stage and is a prognostic factor of poor overall survival

To further investigate the correlation between periostin expression and pancreatic cancer progression, we used TMAs to study periostin expression levels in pancreatic cancer and corresponding paired normal tissues. We performed immunohistochemical staining for periostin on a large cohort of primary pancreatic cancer patients (*n* = 100). Among the 100 patients, both cancer tissues and matched normal tissues were available for 80 patients, whereas only cancer tissues were available for the remaining 20. The clinicopathologic characteristics of 80 PDAC patients and their relationship with periostin expression level are listed in Table 1.

**Table 1:** Clinicopathologic correlations of periostin expression in 80 pancreatic cancer patients

Parameters	High expression (*n* = 64)	Low expression (*n* = 16)	*P*
**Gender**			
Male	37	11	0.424
Female	27	5	
**Age**			0.502
< 60	30	9	
≥ 60	34	7	
**pT stage**			0.019
T1	6	6	
T2	39	8	
T3	19	2	
**pN stage**			0.001
N0	11	12	
N1	28	2	
N2	21	1	
N3	4	1	
**Distant metastases**			0.035
No	38	14	
Yes	26	2	
**Perineural invasion**			0.003
No	25	13	
Yes	39	3	
**Tumor volume (cm^3^)**	34.6 (2.3, 312.7)	18.5 (1.2, 230)	0.005

**Figure 2:**
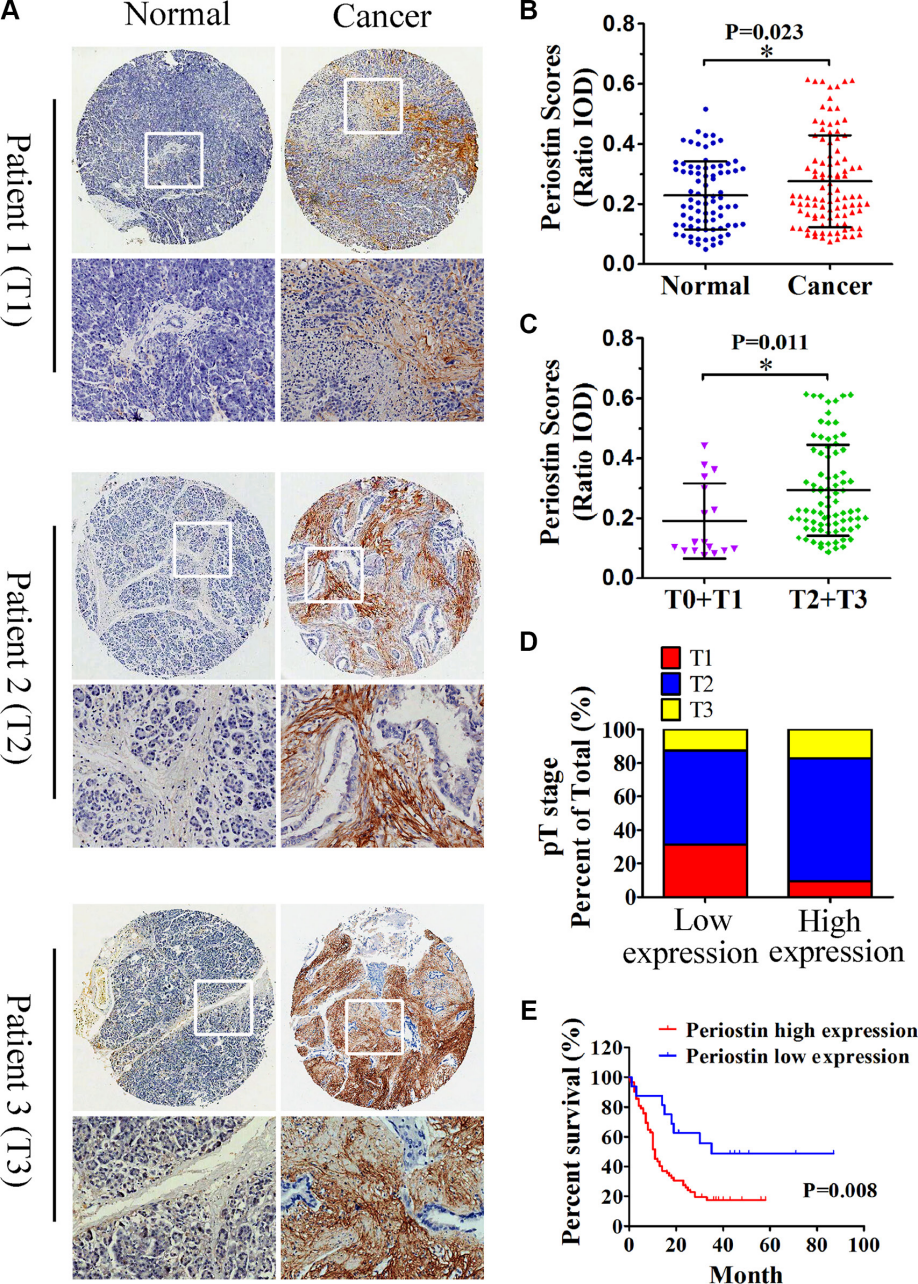
Increased periostin expression correlates with pancreatic cancer progression and poor patient survival (**A**) Immunohistochemical staining of pancreatic cancer and matched normal tissues with anti-periostin antibody. A total of 100 patient samples were stained and representative patient samples of clinical stages T1, T2, and T3 are shown. (**B**) Quantitative analysis of periostin staining in 80 normal tissues and 100 cancer samples showed notably higher staining intensity in pancreatic cancer samples compared with matched normal tissues (IOD, integral optical density). (**C**) Periostin staining intensity according to the clinical stage of pancreatic cancer samples (*n* = 100). (**D**) Upregulated periostin expression positively correlated with the clinical stage of pancreatic cancer (*n* = 16 in the low-expression group, *n* = 64 in the high-expression group). (**E**) High intensity of periostin immunostaining was significantly associated with poor survival. Data are shown as means ± SD. ^*^P < 0 .05.

Semiquantitative analysis showed an increased intensity of periostin staining in pancreatic cancer compared with normal tissues (Figure 2A and 2B). Periostin expression was positively correlated with the clinical stage of pancreatic cancer (Figure 2C and 2D). Moreover, increased periostin expression was significantly associated with shortened patient survival (*P* = 0.008); the median survival rates for patients with high and low periostin expression were 11% and 35%, respectively (Figure 2E). Since periostin expression was positively linked to pathologic features, we postulated that periostin deregulation might have a positive effect on PDAC progression and function as a prognostic predictor.

### Periostin promoted proliferation, migration, invasion and clonogenicity of PCCs *in vitro*

**Figure 3:**
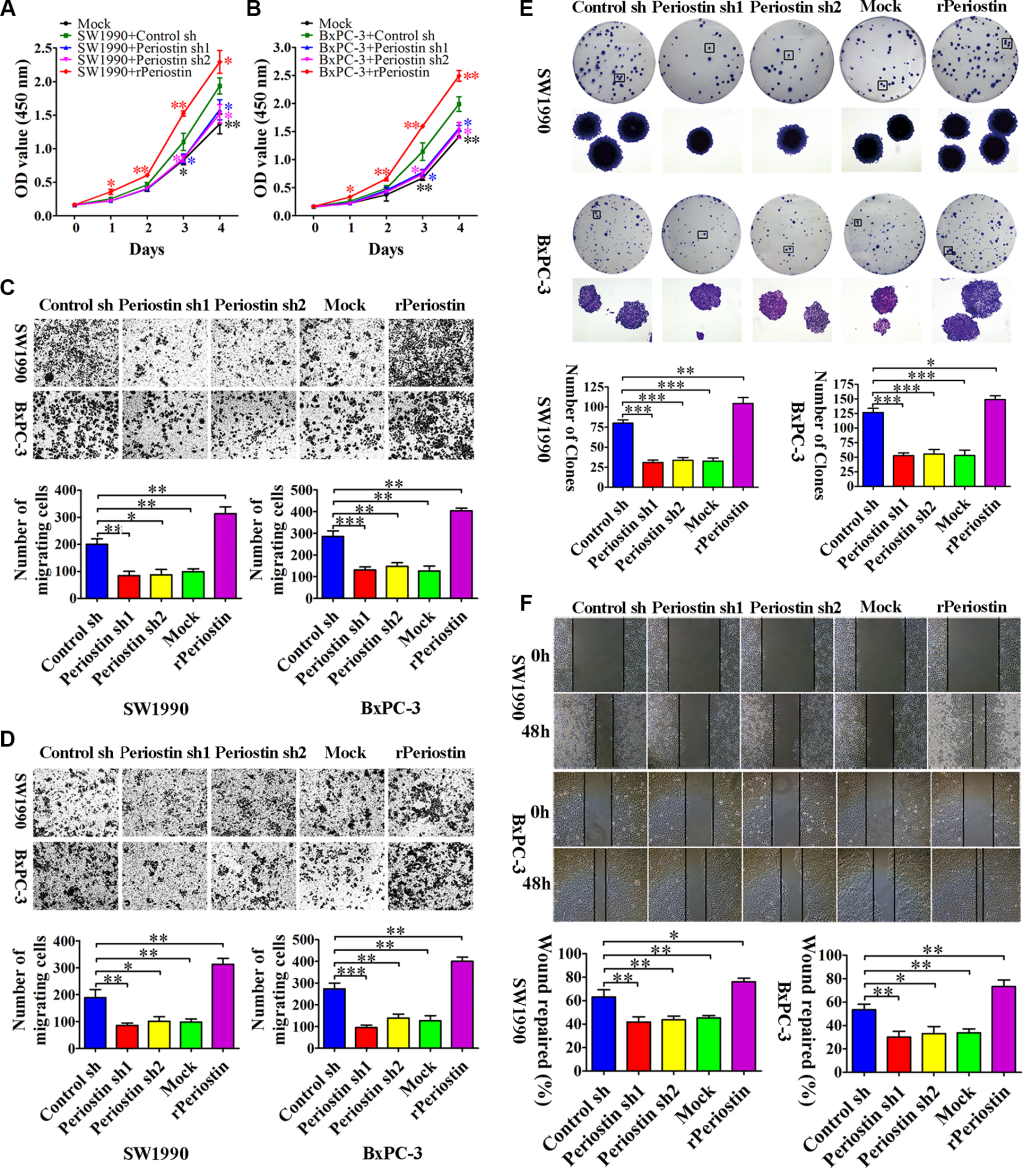
Periostin promotes pancreatic cancer cell proliferation, migration, invasion, and clone formation (**A** and **B**) Periostin knockdown decreased the proliferation rate of SW1990 and BxPC-3 cells. In contrast, increased periostin expression accelerated the proliferation of SW1990 and BxPC-3 cells. OD at 450 nm was measured by CCK-8 assay at 0, 24, 48, 72, and 96 h and is shown as the mean ± SD. (**C** and **D**) Periostin knockdown inhibited migration and invasion of SW1990 and BxPC-3 cells, whereas increased periostin expression exerted the opposite effect. Cells were stained with crystal violet and observed by microscopy (×50 magnification; Zeiss). The number of migration or invasion cells in five random fields was counted by ImageJ software (×100 magnification; Zeiss) and is shown as the mean ± SD. (**E**) periostin knockdown inhibited the ability of SW1990 and BxPC-3 cells to form clonogenic colonies. Cells were stained with crystal violet and photographed without magnification and under light microscopy (×50 magnification; Zeiss). Bar charts show the number of colonies. (**F**) Periostin promoted wound healing in SW1990 and BxPC-3 cells. Cells were scraped with a p10 tip (time 0) and images were captured at the same time every day thereafter (×50 magnification; Zeiss). Migration distance was measured from five fields captured at each indicated time point. The percentage of wound repair for each cell line is shown using bar charts. ^*^P < 0.05, ^**^P < 0.01 and ^***^P < 0.01 vs. control shRNA.

To examine the role of periostin in PCC proliferation, we measured SW1990 and BxPC-3 cell growth by CCK-8 assay after co-culture with shRNA-transfected PSC supernatant or 1 μg/mL rPeriostin. We observed a significant decrease in proliferation of SW1990 and BxPC-3 cells when co-cultured with supernatant of periostin shRNA-transfected PSCs. Moreover, proliferation was markedly increased by co-culture with 1 μg/mL rPeriostin (Figure 3A and 3B). Likewise, rPeriostin led to a significant enhancement of cell migration (Figure 3C), invasion (Figure 3D), and colony formation (Figure 3E), whereas opposite effects were observed after co-culture with periostin shRNA-transfected PSC supernatant. We further analyzed the cell migration ability by a wound-healing assay. As shown in Figure 3F, the rate of wound repair by SW1990 and BxPC-3 cells was significantly suppressed when cells were co-cultured with periostin shRNA-transfected PSC supernatant. In contrast, cell mobility was increased by co-culture with rPeriostin. These data were consistent with the notion that periostin expression is significantly associated with a high degree of pancreatic cancer metastasis.

### Induction of subcutaneous tumors by co-injection of carcinoma cells and pancreatic stellate cells into nude mice

**Figure 4:**
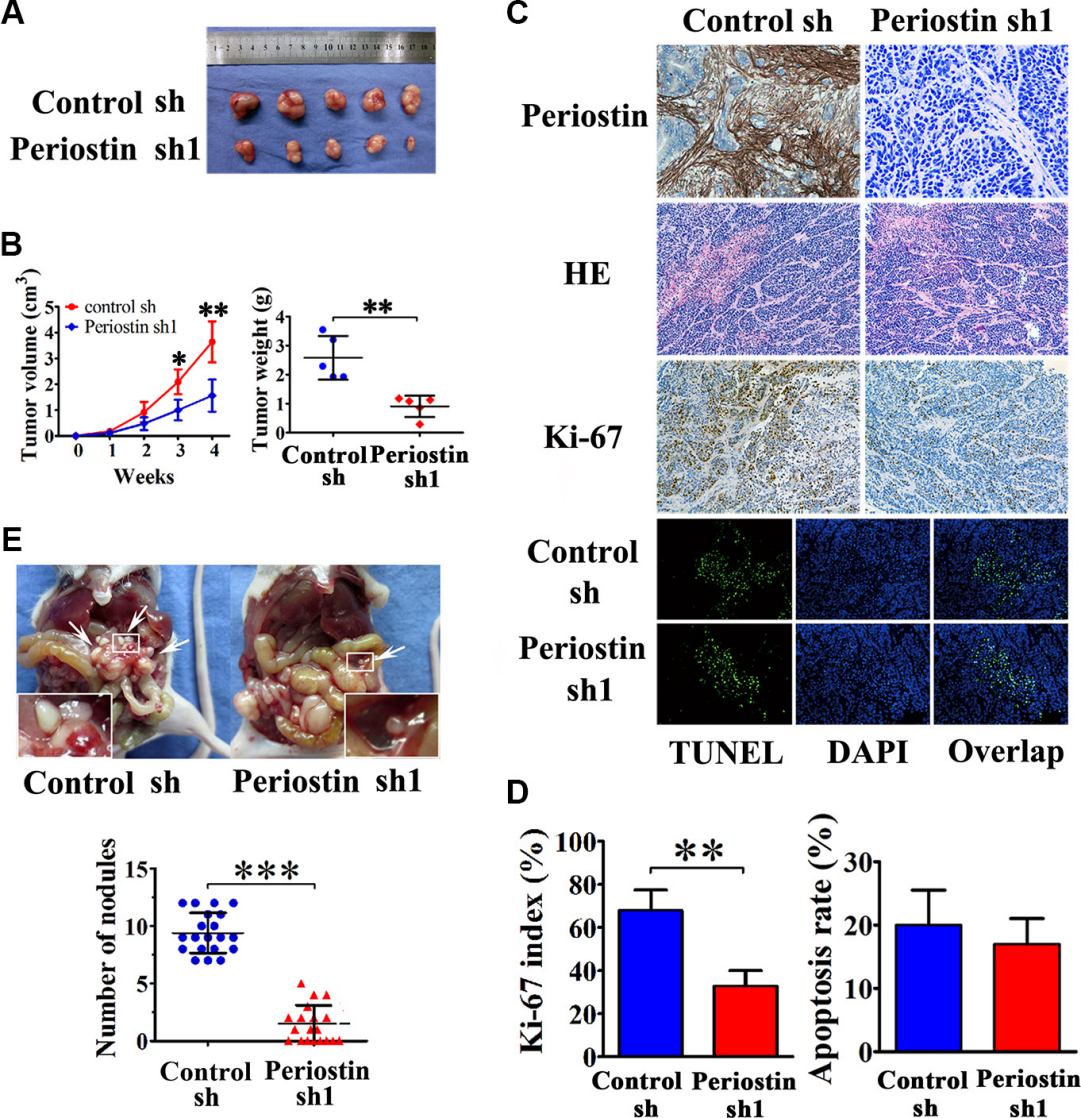
Periostin enhances the tumorigenicity of pancreatic cancer cells *in vivo* and promotes metastasis (**A**) SW1990 cells were co-injected with control shRNA-transfected PSC cells or periostin shRNA1-transfected PSC cells into the right side of nude mice. (**B**) After 4 weeks the mice were sacrificed. SW1990 cells injected with periostin shRNA1-transfected PSCs exhibited slower growth and reduced tumor volumes and weights of xenografts. (**C** and **D**) Immunohistochemical staining showed periostin deposits in the stroma of xenografts, and HE staining revealed that the xenografts had prominent desmoplastic reaction. Xenograft tumors from the periostin-shRNA group contained significantly fewer Ki-67-positive proliferative cells than those from the control group (*n* = 15, five random fields). Periostin expression was not associated with apoptosis in xenograft tumors of SW1990 and PSCs cells as assessed by TUNEL assay. (**E**) Periostin promoted peritoneal metastasis of pancreatic cancer cells in nude mice. SW1990 cells were co-injected with control shRNA-transfected PSCs or with periostin shRNA1-transfected PSCs into the lower-left quadrant of nude mice. Representative pictures are shown; metastatic nodules are marked by white arrowheads. Each group contained 20 mice; analysis was by two-sided unpaired *t*-test.

To verify the role of periostin in pancreatic cancer progression *in vivo*, we performed xenograft tumor assays by subcutaneous co-injection of SW1990 cells and PSC cells that were stably transfected with periostin-shRNA 1 lentivirus. We found that knockdown of periostin in PSCs significantly inhibited tumor growth and reduced tumor volumes and weights of xenografts in nude mice (Figure 4A and 4B). Immunohistochemical staining of periostin showed that periostin was exclusively expressed in the stroma of xenografts, and H&E staining showed that the xenografts exhibited a prominent desmoplastic reaction (Figure 4C). Immunohistochemical staining of Ki-67 showed significantly fewer proliferative cells in periostin-shRNA 1 xenograft tumors (Figure 4C and 4D). Next, we performed TUNEL assays to investigate the effects of periostin on cell apoptosis and observed no significant effect (Figure 4C and 4D). Moreover, periostin promoted abdominal cavity metastasis of pancreatic cancer cells in nude mice (Figure 4E). These data collectively indicate that periostin acts as a novel tumor-promoting gene and positively regulates pancreatic cancer progression.

### Regulation of pancreatic cancer cell activity by periostin is significantly associated with EGFR-Akt and EGFR-Erk-c-Myc signaling pathways

**Figure 5:**
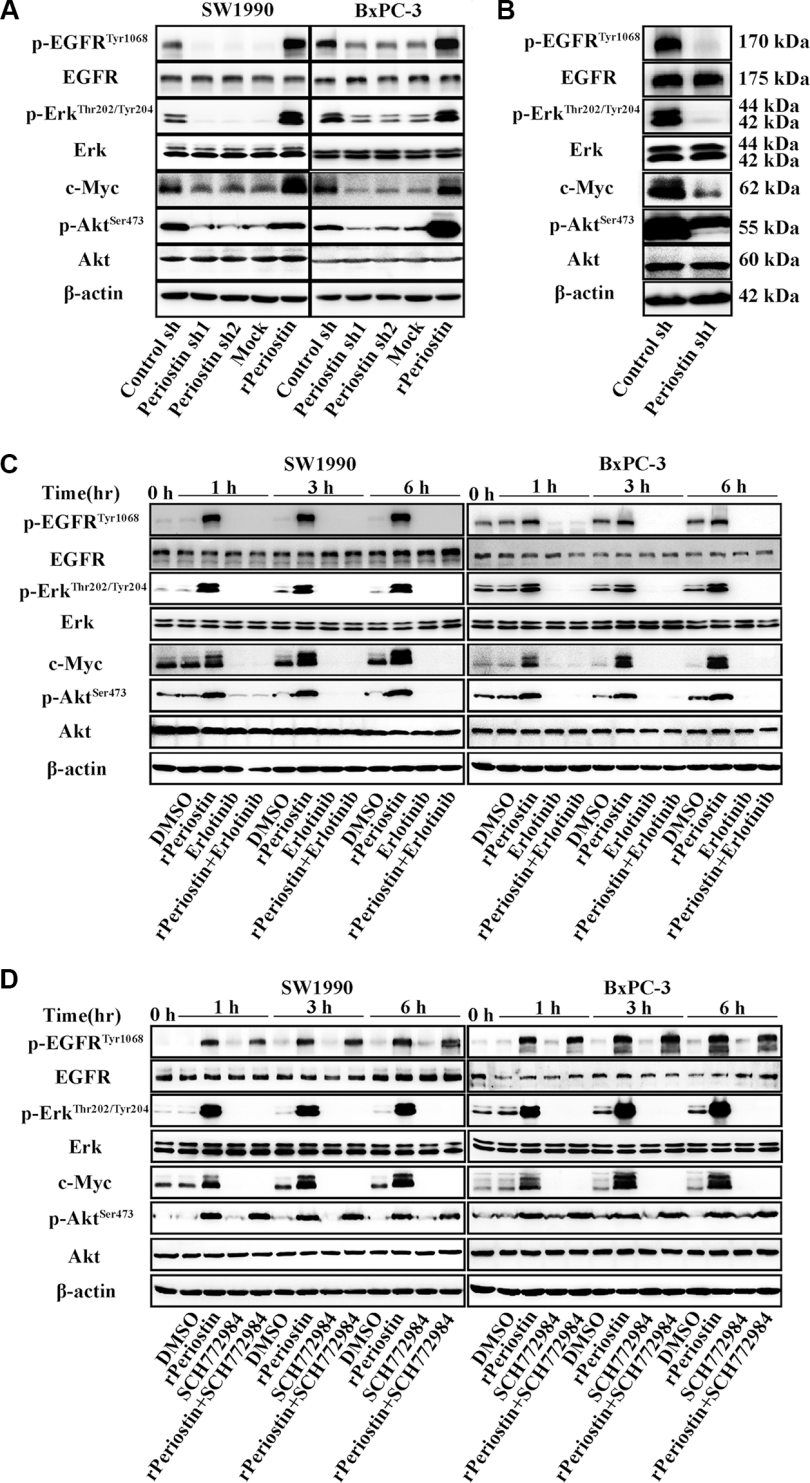
Periostin activates EGFR-Akt and EGFR-Erk-c-Myc signaling to regulate the activity of pancreatic cancer cells (**A**) SW1990 and BxPC-3 cells were treated with the supernatant of control shRNA-transfected PSCs (Control sh), periostin shRNA1-transfected PSCs (Periostin sh1), periostin shRNA2-transfected PSCs (Periostin sh2), DMSO (Mock), or human recombinant protein (rPeriostin). After 12 h, cells were harvested and the basal expression of EGFR, Erk, and their downstream molecules was determined by western blotting. (**B**) Xenograft tumors of nude mice from the control-shRNA group and periostin-shRNA group were also subjected to western blotting using the indicated antibodies. (**C**) SW1990 and BxPC-3 cells were treated with DMSO, rPeriostin, EGFR inhibitor (Erlotinib, 20 μM), or rPeriostin plus Erlotinib. Cells were harvested at 0, 1, 3, and 6 h, and the basal expression of EGFR, Erk, and their downstream molecules was determined by western blotting. (**D**) SW1990 and BxPC-3 cells were treated with DMSO, rPeriostin, Erk inhibitor (SCH772984, 20 μM), or rPeriostin plus SCH772984. Cells were harvested after 0, 1, 3, and 6 h, and the basal expression of EGFR, Erk, and their downstream molecules was determined by western blotting.

To better understand the molecular mechanism by which periostin promotes pancreatic cancer progression, we examined several signaling transduction pathways that might be critical in tumorigenesis and regulated by periostin. Previous reports indicated that EGFR phosphorylation is one of the most common events in pancreatic cancer progression, and we discovered that EGFR phosphorylation was downregulated in periostin-shRNA lentivirus-transfected SW1990 and BxPC-3 cells, whereas the total amount of EGFR was unchanged. We further examined several downstream molecules of p-EGFR and found that p-Akt, p-Erk, c-Myc and were suppressed upon periostin knockdown while the total amounts of AKT and Erk remained unchanged. Moreover, treatment of cells with rPeriostin led to a significant increase in p-EGFR, p-Akt, p-Erk and c-Myc expression levels (Figure 5A). Results from western blot analysis of xenografts of nude mice were consistent with those of the *in vitro* cell experiments (Figure 5B).

**Figure 6:**
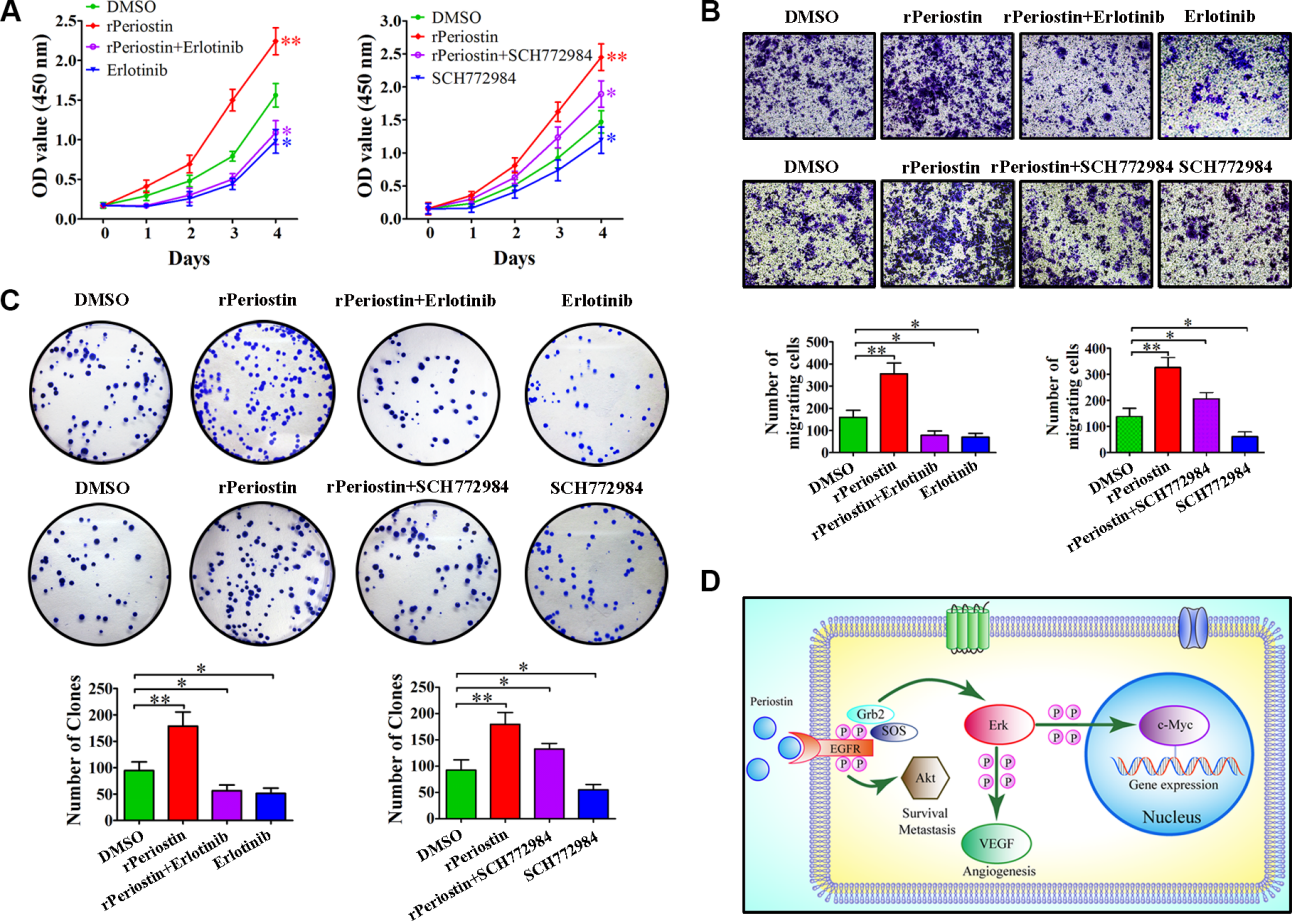
The effects of EGFR and Erk inhibitors on pancreatic cancer cells (**A**) The effect of rPeriostin on promoting growth was completely inhibited by Erlotinib and partially inhibited by SCH772984. (**B**) Migration of SW1990 cells was significantly reduced by treatment with Erlotinib + rPeriostin, but partially reduced by treatment with SCH772984 + rPeriostin. (**C**) Clone formation ability of SW1990 cells was completely inhibited by Erlotinib + rPeriostin and partially inhibited by SCH772984 + rPeriostin. (**D**) Proposed model for the mechanism of action of periostin. Periostin activates EGFR-Akt and EGFR-Erk-c-Myc signaling to regulate survival, metastasis, and gene expression of pancreatic cancer cells.

To further confirm the receptor for periostin and identify the cell signaling pathways that are activated by periostin, we examined the effect of pharmacologic inhibitors of EGFR and Erk phosphorylation on periostin. As shown in Figure 5C and 5D, the effect of periostin on EGFR and Erk phosphorylation was completely abrogated when cells were treated with rPeriostin and the EGFR inhibitor Erlotinib or the Erk inhibitor SCH772984, respectively. Treatment of cells with SCH772984 did not affect EGFR phosphorylation. Moreover, the phosphorylation of downstream molecules was correspondingly inhibited. Next, we examined the effects of these inhibitors on cell function and found that proliferation and clone formation of SW1990 cells were completely inhibited by Erlotinib combined with rPeriostin and partially inhibited by SCH772984 combined with rPeriostin (Figure 6A and 6C). The migration of SW1990 cells was significantly reduced by treatment with Erlotinib combined with rPeriostin, but was reduced less by SCH772984 combined with rPeriostin (Figure 6B). A model for the mechanism of periostin is shown in Figure 6D. Together, our results suggest that periostin regulates the activity of pancreatic cancer cells through EGFR-Akt and EGFR-Erk-c-Myc signaling pathways. Furthermore, the data collectively suggest that EGFR is the receptor for periostin in PDAC.

## DISCUSSION

In recent years, considerable evidence has emerged supporting the notion that the stroma is a critical factor in pancreatic cancer progression [29–31]. The tumor–stroma interaction may be much more complex than previously anticipated and should be reassessed in an unbiased manner [4, 32]. It has now been unequivocally shown that PSCs are the principal effector cells responsible for stroma production [9, 33, 34]. Periostin is abundantly secreted by PSCs and plays a pivotal role in the desmoplastic reaction [4, 20]. Previous studies have shown that periostin is highly expressed in various types of malignant tumor including non-small cell lung cancer [35], breast cancer [36], colon cancer [37], and epithelial ovarian cancer [38]. These studies also demonstrated that periostin is involved in tumor development [20]. To date, there are few studies on the relationship between periostin and pancreatic cancer, and the biological role of periostin has yet to be determined [6, 14, 16]. Therefore, in this study we focused on the expression, biological functions, and potential mechanism of periostin in PDAC tumorigenesis.

Our microarray analysis revealed that periostin was upregulated in the stroma of human PDAC tissues compared with matched adjacent tissues, suggesting that periostin might be a useful diagnostic biomarker for PDAC. In addition, our results showed that periostin was a predictor for advanced clinic stage and shortened overall survival in PDAC patients. We also provide experimental evidence that periostin is exclusively expressed in PSCs. Using CCK-8 assays, transwell assays, scratch tests, and clonality assays, we demonstrated that periostin promoted PCC proliferation, metastasis, invasion, and clonality *in vitro*. Moreover, knockdown of periostin inhibited tumor formation and growth in subcutaneous xenografts, supporting a role for periostin in tumorigenesis *in vivo*. Together, our data prove that periostin activation in PDAC is common and plays an important role in PDAC development. However, the role of periostin in metastasis of various tumors remains conflicted: it appears to play a positive role in metastasis of colon and ovarian cancers but suppresses metastasis in lung and bladder cancers [6]. These differences may be caused by differences in the degree of sensitivity of different cancer cells to periostin or in the concentration of periostin in various tissues.

Several *in vitro* and *in* *vivo* experiments have been conducted to elucidate the intimate interaction between PSCs and PCCs. The tumor-supportive microenvironment of PDAC is created by activation of PSCs by PCCs, and the most potent stimulants for PSCs are platelet-derived growth factor (PDGF), fibroblast growth factor (FGF), and transforming growth factor-β1 (TGF-β1) [8, 34]. Periostin, collagen, fibronectin, and laminin enhance the growth and chemotherapy resistance of PCCs [34]. Moreover, evidence also suggests that periostin creates an autocrine positive-feedback loop on PSCs [6].

A series of recent studies provide insight into the mechanisms by which PSCs influence the activity of PCCs [39, 40]. To better understand the underlying molecular mechanisms by which these processes might be regulated by periostin, we have identified several correlative key signal transduction pathways. In the microenvironment of PDAC, secreted periostin can bind to EGFR to activate various downstream signaling transduction pathways, such as Akt and Erk-c-Myc signaling. Activation of these signaling cascades can promote PCC survival, metastasis and growth. In previous studies, researchers mainly focused on PI3K-Akt and MAPK signaling pathways, which are critical for the growth and motility of PCCs [6]. In addition, activation of the Akt pathway is central to the growth, motility, and energy regulation of PCCs, which increases metastasis and survival even in a nutrient-deprived microenvironment. We found that inhibition of the EGFR pathway by blocking Erk and Akt signaling completely abrogated periostin-mediated EGFR phosphorylation and significantly reduced the expression of c-Myc. Accordingly, the proliferation, metastasis, and colony formation of PCCs decreased greatly. Together, these findings indicate that periostin potentiates metastasis induced by the Akt pathway. In addition, proliferation and clone formation of PCCs were partially inhibited when the Erk pathway was blocked. Erk-c-Myc and Akt pathways are both associated with PCC growth.

In conclusion, our results have shown the biological and clinical significance of periostin in PDAC progression and provide a more comprehensive picture of the underlying molecular mechanisms of periostin. We believe that EGFR and its downstream signals are at least partially responsible for the role of periostin in PDAC progression. Future research into targeted therapies against the tumor microenvironment is necessary. Pharmaceutical intervention to block the interaction of PSCs and PCCs or inhibitory oligonucleotides directed against periostin might have therapeutic potential to suppress PDAC development.

## MATERIALS AND METHODS

### Ethical statement

Informed consent was obtained from all participants and this research was approved by the ethics committee of Shanghai General Hospital affiliated of Shanghai Jiaotong University and performed in accordance with ethical principles. All mouse experiments were manipulated and housed according to the protocols approved by Shanghai Medical Experimental Animal Care Commission.

### Patient samples

Thirty fresh samples of human pancreatic cancer and paired normal tissues were collected during surgery at Shanghai First People's Hospital with the patients’ informed consent. A total of 180-spot, paraffin-embedded tissue array chips (HPan-Ade180Sur-02) including 80 pancreatic cancer tissues and paired normal tissues and 20 cases of tumor tissues only, with 3 to 7 years of follow-up information, were purchased from Shanghai Outdo Biotech, Ltd (Shanghai, China).

### Cell lines and reagents

The human pancreatic cancer cell lines AsPC-1, BxPC3, Capan-1, Capan-2, CFPAC-1, HS766T, Panc-1, and SW1990 were purchased from American Type Culture Collection (Manassas, VA) and normal human pancreatic duct epithelial (HPDE) cells were isolated from normal pancreatic tissues as described [41]. HPDE, Capan-1, Capan-2, HS766T, and Panc-1 were maintained in Dulbecco's modified Eagle medium (DMEM) with 10% FBS (Gibco, Carlsbad, CA). AsPC-1, BxPC-3, HPAF, SW1990 and HUVEC cell lines were maintained in RPMI 1640 with 10% FBS. CFPAC-1 was maintained in IMDM (Iscove's modified Dulbecco's medium) with 10% FBS. Human pancreatic stellate cells (PSCs) were purchased from ScienCell research laboratory (Carlsbad, CA) and maintained in stellate cell medium (ScienCell). The authenticity of the cells was determined by short tandem repeat analysis technology (Cell ID^™^ System, Promega, Madison, WI). All cells were cultured at 37°C in an atmosphere of 5% CO_2_ in air. Human recombinant periostin protein (rPeriostin) was purchased from Biovendor (Heidelberg, Germany) and dissolved in 0.1 M acetate buffer (pH 4) at a concentration of 1 μg/mL. The EGFR inhibitor Erlotinib HCl (OSI-744) and the Erk inhibitor SCH772984 were purchased from Selleck Chemicals (CA, USA), dissolved in 100% DMSO at 10 mM, and stored at −20°C. The final DMSO concentration in the medium was < 0.1% for all experiments.

### Lentivirus transduction for gene silencing

The lentivirus suspension used for shRNA silencing of the periostin gene was purchased from Ebioeasy Ltd (Shanghai, China). The target sequences for periostin were 5′-CGGTGACAGTATAACAGTAAA-3′ named periostin sh1, 5′-CACTTGTAAGAACTGGTATAA-3′ named periostin sh2, respectively. The sequence for scrambled negative control shRNA was 5′-CCTAAGGTTAAGTCGCCCTCG-3′ named control sh. Stable lentivirus transduction was achieved by infection for 48 h and positive cells carrying the GFP fusion protein were selected with puromycin (1 μg/mL) and termed periostin sh or control sh cells. Expression of periostin was measured by western blot analysis (data not shown).

### RNA isolation and quantitative real-time PCR (qRT-PCR)

Total RNA isolation and quantitative real-time PCR were performed according to the manufacturer's instructions. The primers for Periostin were 5′-TGTTGCCCTGGTTATATGAG-3′ (forward) and 5′-ACTCGGTGCAAAGTAAGTGA-3′ (reverse) and those for GAPDH were 5′-GGACCTGACCTGCCGTCTAG-3′ (forward) and 5′-GTAGCCCAGGATGCCCT TGA-3′ (reverse), based on the human periostin and GAPDH cDNA sequences in GenBank. The GAPDH mRNA level was used for normalization. Amplification of each sample was conducted in triplicate. PCR conditions were as follows: 94°C for 15 s, 58°C for 45 s, and 72°C for 20 s, repeated for 35 cycles. Amplified products were separated by 1.0% agarose gel electrophoresis.

### Western blot analysis

Western blot analysis was performed using standard procedures. The primary antibodies used were anti-periostin (1:1,000; ab14041, Abcam, Cambridge, UK), anti-EGFR (1:1,000; sc-71034; Santa Cruz Biotechnology), anti-P-EGFR^Tyr1068^ (1:1,000; #3777; Cell Signaling Technology), anti-Erk (1:1,000; #4695; Cell Signaling Technology), anti-P-Erk^Thr202/Tyr204^ (1:1,000; #9101; Cell Signaling Technology), anti-Akt (1:1,000; #4691; Cell Signaling Technology), anti-P-Akt^Ser473^ (1:1,000; #4060; Cell Signaling Technology), anti-c-Myc (1:500; Santa Cruz Biotechnology), and anti-β-actin (1:5,000; Abcam). The membranes were washed three times in TBST for 10 min each wash and then incubated with goat anti-rabbit IgG horseradish peroxidase-conjugated secondary antibody (Cat. #7074) (1:2,000; Cell Signaling Technology) or horse anti-mouse IgG horseradish peroxidase-linked secondary antibody (Cat. #7074; 1:2,000; Cell Signaling Technology) for 1 h at room temperature. Signals were detected by an enhanced chemiluminescence detection system (Amersham Bioscience, Piscataway, NJ) according to the manufacturer's protocol.

### ELISA

Periostin expression in PSCs and PCCs was measured after 48 h with or without co-culture. The culture supernatants were collected and secreted periostin was quantified by ELISA (Cusabio, Wuhan, China) according to the manufacturer's protocol.

### Cell proliferation assay

Cell proliferation was measured according to the manufacturer's instructions. Briefly, SW1990 and BxPC-3 cells (3 × 10^3^ cells /well) were co-cultured with supernatant from shRNA-transfected PSCs or with 1 μg/mL rPeriostin in 96-well plates. Cell proliferation was examined at 0, 24, 48, 72, and 96 h. After incubation for 2 h at 37°C, the absorbance was measured at 450 nm.

### Migration and invasion assay

Cell migration and invasion were examined according to the manufacturer's instructions. For the migration assay, SW1990 and BxPC-3 cells were incubated in serum-free medium for 24 h and then 3 × 10^4^ cells in 200 μL serum-free medium were added to the upper chamber. For the invasion assay, matrigel (BD Biosciences) was used to simulate the *in vivo* extracellular matrix according to the manufacturer's instructions. Briefly, 6 × 10^4^ SW1990 or BxPC-3 cells in 200 μl serum-free medium were added to the upper chamber, which was precoated with matrigel gel. For both assays, 500 μl of supernatant from shRNA-transfected PSCs or 1 μg/mL rPeriostin was added to the lower chamber as a chemoattractant. Cells were incubated for another 48 h at 37°C and then non-migrating and non-invading cells on the upper surface of the membrane were gently scraped off with cotton swabs. Migrated cells and invasive cells were imaged and counted under a microscope.

### Colony formation assay

A total of 200 SW1990 and BxPC-3 cells were co-cultured with shRNA-transfected PSCs supernatant or 1 μg/mL rPeriostin in six-well plates for 2 weeks. Cell colonies were fixed with 4% methanal for 20 min and stained with 0.04% crystal violet for 20 min. After washing with tap water for 10 min and air drying, colonies were photographed in five random fields and counted using ImageJ software.

### Tumor xenograft model and tumorigenicity assay

A total volume of 100 μl SW1990 cells and periostin sh1 or control sh stably transfected PSCs (3 × 10^6^ cells/mouse) were subcutaneously co-injected into 4-week-old male nude mice (Institute of Zoology, Chinese Academy of Sciences, Shanghai, China). Mice were examined weekly, and tumor nodules were measured with a caliper every week. Tumor volume was evaluated using the following formula: volume = π/6 × (*L* × *W* × *W*), where *L* = the largest tumor diameter and *W* = the smallest tumor diameter. Tumor growth curves were calculated. Nude mice were also co-implanted with SW1990 cells (1 × 10^6^ cells) and periostin sh1 or control sh stably transfected PSCs (1 × 10^6^ cells/mouse) into the lower-left quadrant of the abdomen. Dissemination in the abdominal cavity was evaluated by counting the number of nodules larger than 1 mm in diameter. The two experimental groups were sacrificed after 4 weeks. Finally, all tumor grafts were excised, weighed, harvested, fixed, and embedded in paraffin.

### Immunohistochemistry

To visualize the xenograft tumor, the tumors were dissected and fixed in 4% paraformaldehyde before embedding in paraffin. The tissue was sliced into 4-μm sections and incubated with rabbit anti-human periostin polyclonal antibody and stained with H&E (artificial hematoxylin and eosin stain). Images were captured at ×40 magnification.

A mouse anti-human Ki-67 antigen monoclonal antibody (Dako, dilution 1:50) was used to determine nuclear expression. The Ki-67 index was determined as the mean percentage of cells with Ki-67-positive staining among 1,000 cells. TUNEL staining (Roche, Mannheim, Germany) was used to observe DNA fragmentation of apoptosis by immunohistochemical procedures. All samples were observed using a Nikon microscope (Nikon, Japan). At least five viewing fields containing at least 20 cells were used to obtain one data point.

### Tissue microarray construction

Commercially available tissue microarrays (TMAs) containing a total of 80 pancreatic cancer samples and paired adjacent non-tumor tissues were used in this study (Outdo Biotech, Shanghai, China). All immunohistochemically stained sections were assessed and scored by in-house pathologists. Patients with complete clinicopathologic data were included in the survival analysis.

### Statistical analysis

Statistical comparisons were conducted using Student's test and presented as the mean ± SD, and the log-rank test was used for the patient survival analysis. The association between expression levels of periostin and its related genes in patient samples was analyzed by the Pearson correlation. A *P* value of 0.05 or smaller was considered statistically significant.
